# Associations of *MMP1*, *MMP2* and *MMP3* Genes Polymorphism with Coal Workers’ Pneumoconiosis in Chinese Han Population

**DOI:** 10.3390/ijerph121113901

**Published:** 2015-10-30

**Authors:** Xiaoming Ji, Lijuan Wang, Baiqun Wu, Ruhui Han, Lei Han, Ting Wang, Jingjin Yang, Chunhui Ni

**Affiliations:** 1 Department of Occupational Medicine and Environmental Health, School of Public Health, Nanjing Medical University, Nanjing 211166, China; E-Mails: jxmnjmu@163.com (X.J.); 15250962158@163.com (L.W.); number1989@njmu.edu.cn (B.W.); hanruhui007@163.com (R.H.); hanlei@jscdc.cn (L.H.); wangti08@163.com (T.W.); njmujj@126.com (J.Y.); 2 Institute of Occupational Disease Prevention, Jiangsu Provincial Center for Disease Control and Prevention, Nanjing 210029, China

**Keywords:** genetics, *MMP1*, *MMP2* and *MMP3*, polymorphisms, coal workers’ pneumoconiosis, molecular, epidemiology

## Abstract

Coal workers’ pneumoconiosis (CWP) has been associated with abnormalities in the extracellular matrix remodeling, as well as aberrant matrix metalloproteinases (MMPs) in lung tissues. We investigated the association of three functional polymorphisms in *MMP* gene promoters (*MMP1* rs1799750, *MMP2* rs2285053 and *MMP3* rs522616) with the risk of CWP. A total of 693 CWP cases and 690 controls were included in a case-control study. Genotype analysis was performed by the TaqMan method. Statistically significant differences were found in distributions of *MMP3* rs522616 under a recessive model (*p* = 0.047) between CWP cases and controls. In the stratification analysis, individuals with *MMP3* rs522616 GG genotype decreased the risk of CWP (adjusted OR = 0.72, 95% CI = 0.52–0.99) compared to those with AA/AG genotype obviously, particularly among subgroups of no smokers (adjusted OR = 0.64, 95% CI = 0.41–1.00). Furthermore, serum *MMP3* protein levels measured with enzyme-linked immune-sorbent assay in the control group was significantly lower than that in the CWP groups (*p* = 0.02). Extremely lower *MMP3* among subjects with the rs522616 GG or AG genotype compared with the AA genotype carriers (*p* < 0.05, *p* < 0.01 respectively) in the normal serum. These findings indicate that the *MMP3* rs522616 polymorphism may contribute to the etiology of CWP in the Chinese population and *MMP3* might be a potential diagnostic biomarker for CWP, additional independent studies are warranted to validate our findings in different populations as well as in a larger series.

## 1. Introduction

Coal workers’ pneumoconiosis (CWP) is a chronic occupational lung disease characterized by the pathological accumulation of extracellular matrix (ECM) proteins [1]. It is one of the most widespread occupational lung diseases in China. The exposure to long-term inhalation of coal dust which usually contains free crystalline silica within the lungs [2] can trigger inflammation of the alveoli, eventually resulting in irreversible lung fibrosis and damage [3]. Due to inflammation which induced by coal dust, various proteinases are synthesized and released by activated neutrophils and resident cells, such as macrophages, alveolar epithelial cells and fibroblasts. In addition, the host defense reaction leads to the excessive deposition of ECM, which causes CWP [4]. The pathological processes are often associated with tissue remodeling and elevated turnover of the ECM whereas the precise mechanisms are poorly understood. However, many factors contribute to CWP, including the workplace characteristics and susceptible individuals [5,6,7]. Therefore, identification of new genetic factors for CWP, as well as safer work environment, is needed for strengthening CWP prevention measures.

Matrix metalloproteinases (MMPs) is thought to be important in the maintenance of the ECM and processes of tissue repair. They comprise of more than 20 human zinc-dependent proteolytic enzymes include collagenases (MMP1, MMP8 and MMP13), (MMP3, MMP7 and MMP10) and gelatinases (MMP2 and MMP9). They are involved in degradation of the ECM, leading to tissue remodeling and the development of lung fibrosis [8,9,10]. A variety of MMPs, such as MMP1 (collagenases), MMP2 (gelatinases) and MMP3 (stromelysins), are secreted by monocytes and macrophages and upregulated by cytokines, such as tumour necrosis factor alpha (TNF-α) and interleukin beta (IL-1β). They can degrade fibrillar collagens. Therefore, changes in the levels or activities of these MMPs may play a critical role in the altered collagen metabolism of pulmonary fibrosis. The promoter regions of several *MMP* genes contain biallelic single-nucleotide polymorphisms (SNP) that affect the levels of their gene expression [10,11]. *MMP1* rs1799750 polymorphism has been reported to modify the level of *MMP1* expression and may be associated with a more rapid rate of decline of lung function [12,13]. Hsieh *et* *al*. [14] have addressed the influence of *MMP1* rs1799750 polymorphism on lung function and airway destruction in non-cystic fibrosis bronchiectasis patients in Taiwan. *MMP2* rs2285053 polymorphism has been reported to be involved in extracellular matrix remodeling [15]. Zhou *et* *al*. [16] reported that the rs2285053 CC genotype carriers may relate with a greater risk of lung cancer, compared with non-carriers. *MMP3* has been observed to be a mediator of pulmonary fibrosis, through activation of β-catenin signaling and induction of epithelial-mesenchymal transition [17]. However, it is not known whether any of these polymorphisms are associated with the development of CWP.

In this study, we selected three SNPs of the *MMP* genes which were crucial in lung diseases: *MMP1* rs1799750, *MMP2* rs2285053 and *MMP3* rs522616. The effect of these polymorphisms on *MMP* gene expression has been extensively characterized in previous studies [18,19]. In view of the important roles of *MMPs* have during lung fibrosis and the fact that the functional genetic variations might affect their gene expression, we hypothesized that polymorphisms that lead to increased expression of *MMP* genes or increased activity of the enzymes may determine the development of CWP. Therefore, the aim of this study was to investigate the association of functional promoter polymorphisms in *MMP1*, *MMP2* and *MMP3* genes with CWP. To the best of our knowledge, this is the first study to evaluate the relationship between risk of CWP and functional polymorphisms in *MMP1*, *MMP2* and *MMP3*.

## 2. Materials and Methods

### 2.1. Study Population

A case-control study was performed to examine the association of functionally important gene polymorphisms of *MMP1*, *MMP2* and *MMP3* genes with CWP. Six hundred and ninety-three CWP patients and 690 controls were recruited from the coal mines of Xuzhou Mining Business Group Co., Ltd. (Xuzhou, China) between January 2006 and December 2010, as described previously [20]. In brief, all subjects were underground coal miners and had spent their entire working career with the above mentioned company. Therefore the dust exposure histories between cases and controls were comparable both quantitatively and qualitatively. In addition, occupational health surveillance data including physical examination and chest radiograph were taken every two years for all the underground coal miners, but it was not regular for the retirement. At the same time, high kilovolt chest X-rays were performed for reconfirming the diagnoses based on the China National Diagnostic Criteria for Pneumoconiosis (GBZ 70–2002), which is the same as the 1980 International Labor Organization (ILO) Classification of Pneumoconiosis in the judgment of opacity profusion [21]. Each case was classified as stage I, stage II and stage III according to the size, profusion and distribution range of opacities on chest X-ray by three national certified readers that required agreement at least of two readers. The controls were coal miners and matched with each cases for age (within 5 years), dust exposure period and job type. Each subject received an epidemiological questionnaire on individual information including age, respiratory symptoms, occupational histories, smoking habits and others. The questionnaire was done by face-to-face interviewers and blinded regarding the case or control status of participants. Blood samples (5 mL) were obtained from all subjects, and used for routine lab tests. Written informed consent to participate in this study was obtained from all the individuals. This research protocol was specifically approved by the Institutional Review Board of Nanjing Medical University (Nanjing, China).

### 2.2. Genotyping

In the present study, three SNPs (*MMP1* rs1799750, *MMP2* rs2285053 and *MMP3* rs522616) which might be functional in lung fibrosis diseases were chosen according to the literature. Genotypes for the polymorphisms of *MMP* genes were determined by Taqman Allele Discrimination assay (Applied Biosystems, Carlsbad, CA, USA). Probes and primers used for the genotyping assay were customized as follows: *MMP1* rs1799750 Forward: 5'-GTTTTCTTTCTGCGTCAAGACTGA-3', reverse 5'-CTTGAACTCACATGTTATGCCACTT-3' and probes 5'-ATAAGTCATATCCTTTC[C/-]TAATT-3'; *MMP2* rs2285053 Forward: 5'-GCTGGGTAAAATGAGGCTGAGA-3', reverse 5'-ACCAGTCTTG CCCAATTTCTATCT-3' and probes 5'-CCAGGAG[A/G]GTCCGCAT-3'; *MMP3* rs522616 Forward: 5'-AGAGAGAATTTCAGTCCGGTAAGC-3', reverse 5'-GCCCACGTAGCTGCTCCATA-3' and probes 5'-TGTAATTCATTTCA[G/A]TTCTA-3'. TaqMan PCR was performed in a total volume of 10 µL (3 ng of DNA, 1 × TaqMan master mix, 1 × assay mix) placed in 384-well PCR plates. Fluorescence from PCR amplification was detected using ABI7900 Detector (Applied Biosystems, Carlsbad, CA, USA) and analyzed with the manufacturer’s software. Negative controls were included in each plate to ensure accuracy of the genotyping and approximately 10% of the samples were randomly selected for genotyping in duplicate to monitor genotyping quality and the results were 100% concordant. Genotyping was conducted by two researchers independently in a blinded fashion.

### 2.3. Human MMP3 Levels in Serum Samples

Human *MMP3* protein levels in serum samples were measured by enzyme-linked immune-sorbent assay (ELISA) according to the manufacturer’s instructions (ExCell Biology, Shanghai, China).

### 2.4. Statistical Analysis

Differences in the distributions of demographic characteristics, selected variables, and frequencies of genotypes between the CWP cases and controls were evaluated by the Student’s *t*-test or *χ2*-test. Hardy-Weinberg equilibrium (HWE) was tested using a goodness-of-fit *χ2*-test. The associations between genotypes and CWP were estimated by computing odds ratios (ORs) and their 95% confidence intervals (CIs) from unconditional logistic regression analysis with the adjustment for possible confounders including age, dust exposure period, smoking status and job type. In this study, the dust-exposure cutoff used for the stratified analysis was according to the median of dust-exposure year of the recruited patients and controls. *MMP3* expression was expressed as mean ± standard deviation. One-way analysis of variance (ANOVA) was used to test differences of means between the groups. All statistical tests were two-sided at a significance level of 0.05 and were analyzed using the SAS software (version 9.1; SAS Institute, Inc., Cary, NC, USA). The linkage disequilibrium was analyzed by the SHEsis software (http://analysis.bio-x.cn/SHEsisMain.htm).

## 3. Results

### 3.1. Characteristics of the Study Population

The frequency distributions of the selected characteristics of the cases and controls are presented in Table 1. There was no significant difference in the distribution of age (*p* = 0.113), exposure years (*p* = 0.107), and work types (*p* = 0.534) between the cases and controls. The smoking status of CWP was similar to the controls (*p* = 0.270), but the pack-years smoked in CWP cases was significantly less than that in controls (*p* < 0.001). The frequency distributions and means of the selected characteristics were matched adequately between cases and controls. In addition, of the 686 CWP cases, 415 (59.9%) were stage I, 217 (31.3%) were stage II and the remaining 61 (8.8%) were stage III.

**Table 1:** Demographic and selected variables among the CWP cases and control subjects.

Variables	CWP (*n* = 693)		Controls (*n* = 690)		*p*	
	N	%	N	%	
Age, Year (mean ± SD)	68.0 ± 11.1			67.1 ± 8.4	0.113
Exposure years (mean ± SD)	26.6 ± 9.0			27.3 ± 7.8	0.107
Smoking status					
Never	340	49.1	359	52.0	0.270
Ever	353	50.9	331	48.0	
Former	162	23.4	91	13.2	
Current	191	27.5	240	34.8	
Pack-years smoked					<0.001
0	340	49.1	359	52.0	
0–20	220	31.7	130	18.9	
>20	133	19.2	201	29.1	
Work type					0.534
Tunnel and coal mining	659	95.1	648	94.0	
Transport	16	2.3	17	2.5	
Others	18	2.6	25	3.6	
Stage					
I	415	59.9			
II	217	31.3			
III	61	8.8			

### 3.2. Associations between the Functional MMP Genes Polymorphisms and CWP Risk

The primary information and allele frequencies observed are listed in Table 2. The overall observed distribution of homozygotes and heterozygotes for each polymorphism is consistent with Hardy-Weinberg equilibrium. The minor allele frequency (MAF) of all the 3 SNPs was consistent with that reported in the HapMap database (http://www.hapmap.org).

**Table 2:** Primary information of genotyped SNPs.

Gene	rs No.	Location	Base Change	MAF		HWE ^a^	Genotyping Rate (%)
				Case	Control		
*MMP1*	rs1799750	Promoter	G/-	0.359	0.324	0.663	99.3
*MMP2*	rs2285053	Promoter	C > T	0.225	0.236	0.596	99.2
*MMP3*	rs522616	Promoter	A > G	0.340	0.372	0.117	98.7

^a^ Hardy-Weinberg equilibrium (HWE) *p* value in the control group.

For *MMP2* rs2285053 polymorphism, we did not find any significant association with CWP, being with quite similar allelic and genotypes frequencies in CWP groups and controls, whereas we found that the *MMP1*rs1799750 1G allele was significantly more frequent in CWP patients than in controls (0.364 *vs*. 0.324, *p* = 0.027), suggesting an increased risk of CWP (OR = 1.20, 95% CI = 1.02–1.40). The genotype frequencies of rs1799750 polymorphism (Table 3) were significantly different between the cases and controls under an additive model (*p* = 0.026). For *MMP3* rs522616 polymorphism, significant difference was found in the genotype frequencies under a recessive model (*p* = 0.047). Multivariate logistic regression analyses revealed that a significantly decreased risk was associated with the GG genotype (*p* = 0.041) compared with AA/AG genotype. However, these significances disappeared after the Bonferroni correction.

**Table 3:** Distributions of genotypes of and their associations with risk of CWP.

Variables	CWP Cases		Controls		*p* ^a^	OR (95% CI)	OR (95% CI) ^b^
	N	%	N	%			
rs1799750	*n* = 690		*n* = 683				
2G/2G	276	40.0	309	45.2	0.082	1.00	1.00
2G/1G	325	47.1	305	44.7		1.19 (0.95–1.49)	1.19 (0.95–1.49)
1G/1G	89	12.9	69	10.1		1.44 (1.01–2.06)	1.45 (1.02–2.07)
2Gallele	877	63.6	923	67.6		1.00	
1Gallele	503	36.4	443	32.4	0.027	1.20 (1.02–1.40)	1.20 (1.02–1.40)
ADD					0.026	1.20 (1.02–1.41)	
DOM					0.050	1.24 (1.00–1.54)	1.24 (1.00–1.54)
REC					0.104	1.32 (0.94–1.84)	1.32 (0.94–1.85)
rs2285053	*n* = 690		*n* = 682				
CC	409	59.3	395	57.9	0.684	1.00	1.00
CT	252	36.5	252	37.0		0.97 (0.77–1.21)	0.98 (0.79–1.23)
TT	29	4.2	35	5.1		0.80 (0.48–1.33)	0.79 (0.47–1.32)
C allele	1070	77.5	1042	76.4		1.00	
T allele	310	22.5	322	23.6	0.477	0.94 (0.78–1.12)	0.94 (0.79–1.13)
ADD					0.468	0.94 (0.78–1.12)	
DOM					0.610	0.94 (0.76–1.17)	0.96 (0.77–1.19)
REC					0.414	0.81 (0.49–1.34)	0.80 (0.48–1.32)
rs522616	*n* = 690		*n* = 675				
AA	301	43.6	276	40.9	0.130	1.00	1.00
AG	309	44.8	296	43.8		0.96 (0.76–1.20)	0.96 (0.76–1.20)
GG	80	11.6	103	15.3		0.71 (0.51–0.99)	0.70 (0.50–0.98)
A allele	911	66.0	848	62.8		1.00	
G allele	469	34.0	502	37.2	0.081	0.87 (0.74–1.02)	0.86 (0.74–1.01)
ADD					0.086	0.87 (0.75–1.02)	
DOM					0.307	0.89 (0.72–1.11)	0.89 (0.72–1.10)
REC					0.047	0.73 (0.53–0.99)	0.72 (0.52–0.99)

^a^ two-sided *χ^2^* test; ^b^ adjusted for age, exposure years, working status, and pack-years of smoking in logistic regression model. ADD: additive model; DOM: dominant model; REC: recessive model.

In further stratification analysis for the *MMP3* rs522616 (Table 4), we found that individuals with GG genotype had an obviously decreased risk of CWP (adjusted OR = 0.72, 95% CI = 0.52–0.99) than those with AA/AG genotype. This decreased risk was also more pronounced among subgroups of no smokers (adjusted OR = 0.64, 95% CI = 0.41–1.00) and CWP patients with stage I (adjusted OR = 0.66, 95% CI = 0.45–0.96).

**Table 4:** Stratification analyses between the genotypes of *MMP3* rs522616 and CWP risk.

Variables	Cases/Controls	Genotypes (Cases/Controls)				*p* ^a^	OR (95% CI) ^a^
		AA/AG		GG			
		*n*	%	*n*	%		
Total age	690/675	610/572	88.4/84.7	80/103	11.6/15.3	0.041	0.72 (0.52–0.99)
<68	275/397	246/335	89.4/84.4	29/62	10.6/15.6	0.199	0.72 (0.43–1.19)
≥68	415/278	364/237	87.7/85.2	51/41	12.3/14.8	0.379	0.82 (0.52–1.28)
Exposure years							
<27	268/261	236/216	88.1/82.8	32/45	11.9/17.2	0.060	0.62 (0.38–1.02)
≥27	422/414	374/356	88.6/86.0	48/58	11.4/14.0	0.268	0.79 (0.52–1.20)
Smoking status							
Never	339/351	302/295	89.1/84.0	37/56	10.9/16.0	0.049	0.64 (0.41–1.00)
Ever	351/324	308/277	87.8/85.5	43/47	12.2/14.5	0.344	0.81(0.52–1.26)
Stage							
I	413/675	368/572	89.1/84.7	45/103	10.9/15.3	0.032	0.66 (0.45–0.96)
II	216/675	193/572	89.4/84.7	23/103	10.6/15.3	0.213	0.73 (0.44–1.20)
III	61/675	49/572	80.3/84.7	12/103	19.7/15.3	0.188	1.59 (0.80–3.18)

^a^ adjusted for age, exposure years, job type, and pack-years of smoking in logistic regression model.

**Figure 1:**
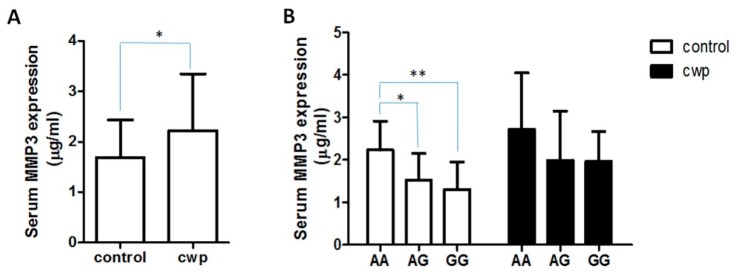
*MMP3* expression (mean ± SD) in normal and CWP serum samples. The *MMP3* protein expression in serum samples from 36 CWP patients and 36 normal subjects was measured by enzyme-linked immune-sorbent assay (ELISA). (**A**) Serum *MMP3* levels were significantly lower in with healthy individuals than those in CWP patients (1.68 ± 0.75 *vs*. 2.22 ± 1.12 μg/mL, *p* = 0.02); (**B**) In the normal serum, there were significantly lower *MMP3* among subjects with the rs522616 GG or AG genotype compared with the AA genotype carriers (1.30 ± 0.64 *vs*. 2.23 ± 0.68 μg/mL, *p* < 0.05; 1.52 ± 0.63 *vs*. 2.23 ± 0.68 μg/mL, *p* < 0.01, respectively. *n* = 12 in each group). However, no statistically significant differences of *MMP3* expression were found between GG, AG and AA genotypes in CWP serum samples (1.98 ± 0.70, 2.00 ± 1.16 and 2.72 ± 1.33 μg/mL. *n* = 12 in each group). * *p* < 0.05, ** *p* < 0.01.

### 3.3. Association and Stratification Analysis between MMP3 Expression and CWP Risk

As shown in Figure 1, the *MMP3* protein expression in serum samples from 36 CWP patients and 36 normal subjects were detected and analyzed. Serum *MMP3* levels were significantly lower in healthy individuals than those in CWP patients (1.68 ± 0.75 *vs*. 2.22 ± 1.12 μg/mL, *p* = 0.02, Figure 1A). In the normal serum, there were significantly lower *MMP3* among subjects with the rs522616 GG or AG genotype compared with the AA genotype carriers (1.30 ± 0.64 *vs*. 2.23 ± 0.68 μg/mL, *p* < 0.05; 1.52 ± 0.63 *vs*. 2.23 ± 0.68 μg/mL, *p* < 0.01, respectively. *n* = 12 in each group, Figure 1B). However, no statistically significant differences of *MMP3* expression were observed between GG, AG and AA genotypes in CWP serum samples (1.98 ± 0.70, 2.00 ± 1.16 and 2.72 ± 1.33 μg/mL. *n* = 12 in each group, Figure 1B).

## 4. Discussion

In this present case-control study, three functional polymorphisms in the *MMP1*, *MMP2*, and *MMP3* genes were investigated in regard to an association with risk of CWP in a Chinese population. We found that *MMP3* rs522616 in the promoter region was significantly associated with CWP, and the association was more dramatic in subgroups of no smokers and CWP patients with stage I. Furthermore, *MMP3* protein levels in serums within the control group were significantly lower than those in the CWP groups. To the best of our knowledge, this is the first study on the association of functional polymorphisms in the *MMP1*, *MMP2*, and *MMP3* genes with CWP risk.

CWP is a chronic lung fibrosis disease characterized by proliferation of fibroblasts and excessive exposition of extracellular matrix. Genetic factors such as polymorphisms can contribute to the development of CWP. Many genetic studies in CWP patients have been reported [6,21,22]. *MMP* genes play a vital role in tissue remodeling process and degradation of the component of ECM.

In this study, we found that *MMP1* rs1799750 1G increased the risk of developing CWP. Wang and his colleagues reported that the frequency of *MMP1* rs1799750 polymorphism was associated with lung fibrosis and lung destruction in TB patients [23]. 1G genotype of *MMP1* rs1799750 polymorphism was reported to independently increase the risk for the development of moderate and advanced pulmonary fibrosis by 5- and 10-fold, respectively [23]. High levels of *MMP1* expression in monocytes cells have been seen in cells from subjects with the 1G allele, but not the 2G/2G genotype. Upregulation of *MMP1* gene and protein expression has been shown in human lung fibrosis [24,25]. Fukuda *et* *al*. [26] demonstrated that increased *MMP1* in epithelial cells in areas of intra-alveolar fibrosis in biopsy specimens from idiopathic pulmonary fibrosis patients. These reports suggest that *MMP1* polymorphisms with upregulated *MMP1* activity may be associated with fibrogenesis.

In addition, we demonstrated that the GG genotype of rs522616, which is located in the promoter region of *MMP3*, was associated with a lower risk of developing CWP. It is possible that a variant in the promoter region of *MMP3* could affect the production of proteolytic enzymes. Meanwhile, it may have an effect on the risk of CWP occurrence. Huai *et* *al*. [27] have reported that the transcription factor C-MYB can bind to rs522616 A allele of the *MMP3* promoter, activate its transcription and lead to a higher expression of this gene through bioinformatics analyses and ChiP assays in HEK293 and HUVEC cells, demonstrating that rs522626 G allele is related to a lower expression of *MMP3*. It may be the reason that serum *MMP3* expression was lower in subjects with the rs522616 GG or AG genotype compared with the AA genotype carriers in the control group. Emonard *et* *al*. [28] found that matrix from acellular sarcoid granulomas induced increased production of *MMP3* by cultured fibroblasts. Moreover, logistic regression analysis from our data revealed that the variant genotype G was associated with a significantly decreased risk of CWP, suggesting that the *MMP3* rs522616 polymorphism may contribute to the etiology of CWP in the Chinese Han population. Therefore, individuals with *MMP3* rs522616 GG genotype may be not more vulnerable to permanent lung fibrosis due to the decreased *MMP3* expression, with the reduced ability to degrade ECM. Furthermore, we found that the decreased risk associated with the *MMP3* rs522616 GG genotype was more evident among no smokers. The possible explanation is that individuals in this subgroup may be less probably to have been exposed to some risk factors related with the etiology of CWP, such as tobacco smoking [29]. Several association studies have reported that smoking can induce lung fibrosis [30,31]. A variety of MMPs are secreted by monocytes and macrophages and upregulated by cytokines, such as *TNF-α* and *IL-1β*. The A-238 transition in the promoter region of *TNF-α* have been reported to be associated with CWP in Chinese population [32]. The A-238 variant was related to alter *TNF-α* production. Therefore, the A-238 variant may play a critical role in the altered *MMP3* of individuals with different rs522616 genotypes.

Some possible limitations should be mentioned in this study. First, this is a case-control study, the general populations of coal miners could not be represented because of the selection bias in China. Second, our sample size is relatively moderate with limited statistical power, especially for the stratified analysis. Third, we only assessed the association between different genotypes of *MMP3* rs522616 and *MMP3* expression, neglecting other SNPs in *MMP3* and other *MMP* genes. Further studies with larger cohorts to reduce the possibility of false-positive associations arising as a result of low power will be needed to confirm our findings. In addition, functional study should be needed for demonstrating the mechanism underlying the association.

## 5. Conclusions

The present study suggests that *MMP3* rs522616 polymorphism is associated with CWP susceptibility and *MMP3* might be a potential diagnostic biomarker for CWP. Further independent studies with larger cohorts are warranted to validate our findings in different populations.
